# The clinical utility of the urine-based lateral flow lipoarabinomannan assay in HIV-infected adults in Myanmar: an observational study

**DOI:** 10.1186/s12916-017-0888-3

**Published:** 2017-08-04

**Authors:** Swe Swe Thit, Ne Myo Aung, Zaw Win Htet, Mark A. Boyd, Htin Aung Saw, Nicholas M. Anstey, Tint Tint Kyi, David A. Cooper, Mar Mar Kyi, Josh Hanson

**Affiliations:** 1 University of Medicine 2, Yangon, Myanmar; 2 Insein General Hospital, Yangon, Myanmar; 3 University of Adelaide, Lyell McEwin Hospital, Adelaide, Australia; 4 The Kirby Institute, University of New South Wales, Sydney, Australia; 5 Menzies School of Health Research, Charles Darwin University, Darwin, Australia; 6 Department of Medical Care, Ministry of Health, Nay Pyi Taw, Myanmar

**Keywords:** Human immunodeficiency virus, Tuberculosis, Diagnostic test, Clinical management, Myanmar, Lipoarabinomannan

## Abstract

**Background:**

The use of the point-of-care lateral flow lipoarabinomannan (LF-LAM) test may expedite tuberculosis (TB) diagnosis in HIV-positive patients. However, the test’s clinical utility is poorly defined outside sub-Saharan Africa.

**Methods:**

The study enrolled consecutive HIV-positive adults at a tertiary referral hospital in Yangon, Myanmar. On enrolment, patients had a LF-LAM test performed according to the manufacturer’s instructions. Clinicians managing the patients were unaware of the LF-LAM result, which was correlated with the patient’s clinical course over the ensuing 6 months.

**Results:**

The study enrolled 54 inpatients and 463 outpatients between July 1 and December 31, 2015. On enrolment, the patients’ median (interquartile range) CD4 T-cell count was 270 (128–443) cells/mm^3^. The baseline LF-LAM test was positive in 201/517 (39%). TB was confirmed microbiologically during follow-up in 54/517 (10%), with rifampicin resistance present in 8/54 (15%). In the study’s resource-limited setting, extrapulmonary testing for TB was not possible, but after 6 months, 97/201 (48%) with a positive LF-LAM test on enrolment had neither died, required hospitalisation, received a TB diagnosis or received empirical anti-TB therapy, suggesting a high rate of false-positive results. Of the 97 false-positive tests, 89 (92%) were grade 1 positive, suggesting poor test specificity using this cut-off. Only 21/517 (4%) patients were inpatients with TB symptoms and a CD4 T-cell count of < 100 cells/mm^3^. Five (24%) of these 21 died, three of whom had a positive LF-LAM test on enrolment. However, all three received anti-TB therapy before death — two after diagnosis with Xpert MTB/RIF testing, while the other received empirical treatment. It is unlikely that knowledge of the baseline LF-LAM result would have averted any of the study’s other 11 deaths; eight had a negative test, and of the three patients with a positive test, two received anti-TB therapy before death, while one died from laboratory-confirmed cryptococcal meningitis. The test was no better than a simple, clinical history excluding TB during follow-up (negative predictive value (95% confidence interval): 94% (91–97) vs. 94% (91–96)).

**Conclusions:**

The LF-LAM test had limited clinical utility in the management of HIV-positive patients in this Asian referral hospital setting.

**Electronic supplementary material:**

The online version of this article (doi:10.1186/s12916-017-0888-3) contains supplementary material, which is available to authorized users.

## Background

Tuberculosis (TB) is the most common cause of death in HIV-infected patients globally [1, 2]. Limited access to laboratory services in low- and middle-income countries means that almost half the fatal cases of TB/HIV co-infection are unrecognised before the patients die [1, 3].

Even where there is access to laboratory services, there are significant challenges in diagnosing TB in HIV-positive patients [4]; mycobacterial culture is resource-intensive and may take weeks to provide results, sputum microscopy is rapid and inexpensive but has a sensitivity of less than 50% in HIV-positive patients [5], and while the Xpert MTB/RIF assay is endorsed by the World Health Organization (WHO), its use has not been shown to improve mortality [6–8].

Against this background, the development of the point-of-care urine-based lateral flow lipoarabinomannan (LF-LAM) test has been hailed as a significant advance [9]. The test requires no laboratory infrastructure, is cheaper and simpler to perform than the Xpert MTB/RIF assay, and lacks the infection risks associated with sputum collection [10]. The test has shown greatest utility in facilitating the diagnosis of TB in HIV-positive hospital inpatients with advanced immunodeficiency and symptoms of TB, the population at greatest risk of death [4, 11]. In sub-Saharan Africa, the use of LF-LAM testing to diagnose TB proved cost-effective [12, 13] and reduced 8-week mortality in HIV-positive hospital inpatients [14]. More data are required to support the use of the test in outpatients, although extrapolating from the findings in inpatients, it has been suggested that it may have a role in seriously ill outpatients with more advanced immunodeficiency [10].

The utility of LF-LAM in excluding the diagnosis of TB in HIV-positive patients is incompletely defined, although the expert consensus in current WHO recommendations is that the test should not be used for this purpose [10]. The test’s relatively high negative predictive value (NPV) might be hypothesised to assist in excluding TB before isoniazid prophylaxis therapy (IPT) or antiretroviral therapy (ART) is initiated, but the safety of this approach has not been established. It is also not clear that using the test to exclude TB is better than a simple clinical history, which also has an excellent NPV [15].

Importantly, most of the research to define the clinical utility of the LF-LAM test has been performed in sub-Saharan Africa, the region with the world’s highest rates of HIV/TB co-infection. By contrast, relatively few data have been collected in Asia [2, 16–18]. This is significant as the performance of a diagnostic test depends on the local prevalence of the condition, and effective management strategies based on the performance of a diagnostic test in one location are not necessarily generalisable to other populations [19].

Furthermore, the LF-LAM test itself has evolved over time [20]. When the faintest bands in an earlier version were used to define a positive result (grade 1 cut-off) as recommended by the manufacturer, the test’s specificity in culture-negative patients was poor (66%, 95% confidence interval (CI), 57–74); however, this improved to 96% (95% CI, 89–100) when the more intense grade 2 cut-off was used [21]. To simplify the test’s interpretation, in January 2014, the manufacturer revised the reference card that ‘scores’ the test to have only four bands; the band intensity for a grade 1 positive test on the new reference card corresponding to the band intensity of the previous grade 2 positive test. However, the performance of this revised version of the test requires validation [4].

Therefore, this study was performed to evaluate the clinical utility of the LF-LAM test in a cohort of HIV-positive patients in Myanmar. It was hypothesised that the test result would correlate well with the patients’ subsequent clinical course, providing justification for its employment in diagnostic and management strategies to reduce mortality in HIV-positive patients in Southeast Asian countries.

## Methods

**Fig. 1:**
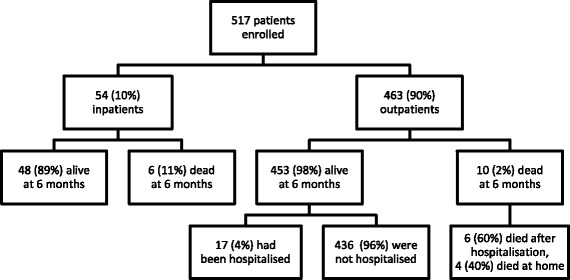
Study profile. No patients were lost to follow-up during the study period

This observational study was performed at Insein General Hospital, a tertiary referral hospital in Yangon, Myanmar’s largest city. All HIV-positive inpatients and outpatients (whether seeking care for symptoms or in routine follow-up) were eligible for inclusion. Patients were enrolled consecutively between July 1, 2015, and December 31, 2015 (Fig. 1). A 6-month period was chosen to assess the everyday applicability of the LF-LAM test in this high caseload hospital.

After obtaining informed consent, study doctors — who were not involved in the patient’s routine care — collected a detailed medical history using a dedicated pro forma, performed a physical examination and recorded available laboratory data. Patients were defined as having symptoms suggestive of TB if they had experienced any cough, fever, night sweats or weight loss in the previous month. A chest X-ray was ordered and sputum was collected for culture (Lowenstein Jensen medium) and Xpert MTB/RIF testing in all patients. If the patient could not produce sputum spontaneously, it was induced using hypertonic saline with appropriate precautions. A separate study doctor, blinded to the clinical details of the case, then collected urine in all patients and performed the LF-LAM test (Alere Determine™ TB LAM Ag) according to the manufacturer’s instructions. Positive tests were graded according to the manufacturer’s new reference card (grade 1, 2, 3 or 4). For the test result to be scored as grade 1, the band on the test-strip had to be at least as intense as the corresponding grade 1 band on the reference card, but not as intense as the grade 2 band. If the study doctor was uncertain of the LF-LAM result, a consensus was reached with another study doctor and one of the authors (SST), both of whom were also unaware of the patient’s clinical presentation. To further reduce the risk of bias, digital photographs were taken of the LF-LAM tests at the time of testing. Another author (JH), unaware of the patient’s clinical presentation, later examined these photographs to grade the tests independently.

The patient’s usual medical team continued the patient’s care without knowledge of the LF-LAM test result, as the test was not approved for use in Myanmar at the time the study was undertaken. This care included the initiation of empirical TB therapy if this was felt to be clinically indicated. The patients’ clinical course was followed prospectively by the study doctors for 6 months after enrolment. The patients were defined as having confirmed TB if they returned either a positive culture for *Mycobacterium tuberculosis* or a positive Xpert MTB/RIF assay in the ensuing 6 months. Patients were defined as having a complicated course if they had a confirmed TB diagnosis, received empirical anti-TB therapy, required hospitalisation or died during the study.

Extrapulmonary samples were not collected, as facilities to process these samples were not available in Myanmar’s public health system at the time of the study. However, it was reasoned that, after 6 months of follow-up, if a patient had not had a confirmed TB diagnosis and had not died, required hospitalisation or received empirical anti-TB therapy, a positive baseline LF-LAM test was likely to be a false-positive test.

### Statistical analysis

All data were de-identified and entered into an electronic database (Microsoft Excel). Statistical analysis was performed using statistical software (Stata). Groups were analysed using the Kruskal–Wallis test and the χ^2^ test. Inter-rater reliability was tested using Cohen’s kappa. The LF-LAM test’s performance was evaluated using the original test results reported by the study doctors rather than the results of the validation photographs.

## Results

### Patient characteristics

**Fig. 2:**
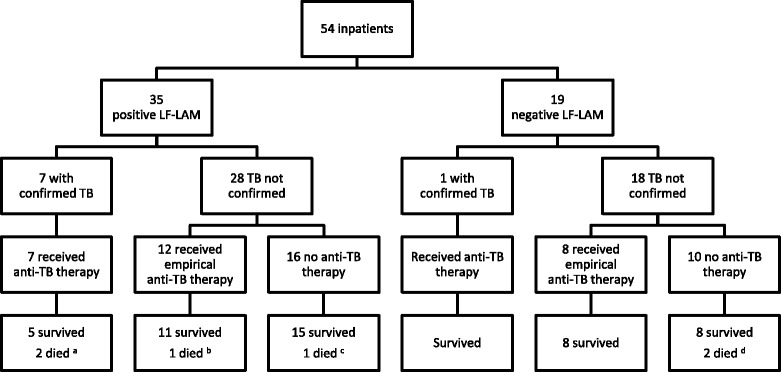
Clinical course of patients enrolled as inpatients. ^a^One case of TB meningitis, one case of miliary TB. ^b^One case of suspected TB meningitis. ^c^One case of microbiologically confirmed cryptococcal meningitis. ^d^Two cases of suspected *Pneumocystis jirovecii* pneumonia

**Fig. 3:**
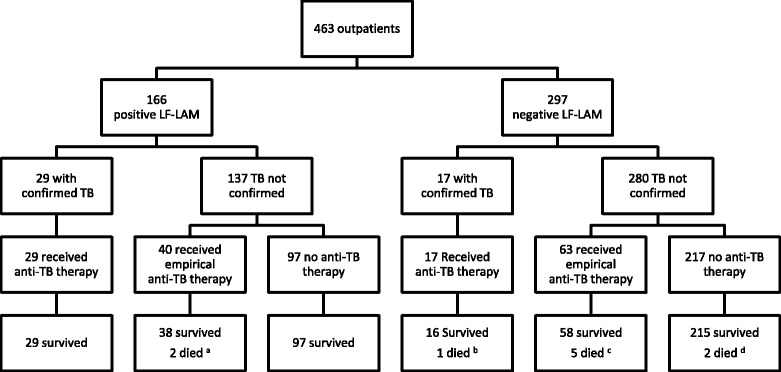
Clinical course of patients enrolled as outpatients. ^a^One case of suspected miliary TB, one case of suspected TB meningitis. ^b^One case of confirmed TB meningitis (rifampicin resistant on Xpert MTB/RIF assay). ^c^Two cases of suspected TB meningitis, one case of suspected miliary TB, one case of suspected toxoplasmosis, one case of HIV cachexia. ^d^One case of confirmed cryptococcal meningitis, one case of suspected toxoplasmosis

The study enrolled 517 consecutive patients (Fig. 1), 463 (89.6%) inpatients (Fig. 2) and 54 (10.4%) outpatients (Fig. 3). The patients’ median age was 34 (interquartile range (IQR), 30–41) years; 259 (50.1%) were male and 258 (49.9%) were female. The median (IQR) CD4 T-cell count on enrolment was 270 (128–443) cells/mm^3^; 360/517 (70%) patients were receiving ART and 14/517 (3%) were receiving IPT. The patients’ other characteristics are presented in Table 1.

**Table 1:** Association between LF-LAM test results on enrolment, patient characteristics and subsequent clinical course

	All patients n = 517	Negative test (Grade 0) n = 316	Positive (≥ Grade 1) n = 201	Positive (≥ Grade 2) n = 43	Positive (≥ Grade 3) n = 20
Age, years	34 (30–41)	34 (30–41)	34 (29–41)	34 (28–42)	34 (28–45)
Male sex	259 (50%)	158 (50%)	101 (50%)	22 (51%)	9 (45%)
Enrolled as inpatient	54 (10%)	19 (6%)	35 (17%)^***^	15 (35%)^***^	10 (50%)^***^
Symptoms of systemic TB ^a^	169 (33%)	90 (29%)	79 (39%)^*^	32 (74%)^***^	16 (80%)^***^
Cough in last month	116 (22%)	56 (18%)	60 (30%)^***^	23 (53%)^***^	12 (60%)^***^
Current cigarette smoker	150 (29%)	88 (28%)	62 (31%)	16 (37%)	8 (40%)
Current tobacco chewing	222 (43%)	133 (42%)	89 (44%)	23 (53%)	11 (55%)
Hazardous alcohol consumption	71 (14%)	31 (10%)	40 (20%)^***^	13 (30%)^***^	6 (30%)^*^
Body mass index	20.8 (18.8–23.1)	21.2 (19.3–23.5)	20.4 (18.6–22.7)^*^	19.5 (17.8–22.0)^**^	19.4 (17.6–22.0)
Recent subjective weight loss	235 (45%)	135 (43%)	100 (50%)	30 (70%)^***^	15 (75%)^**^
Abnormal respiratory examination	28 (5%)	8 (3%)	20 (10%)^***^	11 (26%)^***^	6 (30%)^***^
On anti-retroviral therapy	360 (70%)	225 (71%)	135 (67%)	24 (56%)^*^	9 (45%)^*^
On isoniazid prophylaxis therapy	14 (3%)	9 (3%)	5 (2%)	1 (2%)	0
CD4 T-cell count, cells/mm^3^ ^b^	270 (128–443)	289 (151–481)	233 (95–409)^*^	112 (55–264)^***^	71 (31–159)^***^
Haemoglobin, g/dL^b^	10.5 (9.2–12.2)	10.7 (9.4–12.1)	10.1 (9.0–12.0)	9.1 (8.1–10.2)^***^	8.6 (6.5–10.1)^***^
White cell count, × 10^9^/L^b^	6.9 (5.3–8.7)	6.9 (5.4–8.6)	6.9 (5.3–8.9)	6.3 (5.4–10.5)	6.1 (4.5–6.2)
Lymphocytes, × 10^9^/L^b^	1.7 (1.2–2.5)	1.8 (1.3–2.5)	1.7 (1.1–2.4)	1.3 (0.5–2.2)^**^	0.8 (0.4–1.6)^***^
Platelets, × 10^9^/L^b^	291 (225–357)	288 (219–348)	295 (239–375)	277 (224–330)	276 (216–332)
Creatinine, μmol/L^b^	85 (72–98)	85 (72–97)	86 (73–98)	80 (74–98)	84 (68–114)
Total bilirubin, μmol/L^b^	13.2 (7.8–17.1)	12.6 (7.8–17.3)	13.5 (7.8–16.9)	15.0 (6.7–16.9)	12.5 (5.5–16.2)
Abnormal chest X-ray^b^	186 (38%)	102/294 (35%)	84/190 (44%)^*^	17/40 (43%)	9/18 (50%)
Gross abnormality on chest X-ray^b^	57 (12%)	29/294 (10%)	28/190 (15%)	8/40 (20%)	3/18 (17%)
TB diagnosis at 1 months	37 (7%)	10 (3%)	27 (13%)^***^	14 (33%)^***^	8 (40%)^***^
TB diagnosis at 3 months	44 (9%)	12 (4%)	32 (16%)^***^	16 (37%)^***^	8 (40%)^***^
TB diagnosis at 6 months	54 (10%)	18 (6%)	36 (18%)^***^	17 (40%)^***^	9 (45%)^***^
Empirical TB therapy at 1 month^c^	33 (7%)	17 (6%)	16 (9%)	5 (17%)^*^	1 (8%)
Empirical TB therapy at 3 months^c^	84 (18%)	45 (15%)	39 (23%)^*^	11 (41%)^***^	6 (50%)^**^
Empirical TB therapy at 6 months^c^	123 (27%)	71 (24%)	52 (32%)	13 (50%)^**^	6 (55%)^*^
Dead at 1 month	5 (1%)	3 (1%)	2 (1%)	2 (5%)^*^	1 (5%)
Dead at 3 months	13 (3%)	7 (2%)	6 (3%)	5 (12%)^***^	4 (20%)^***^
Dead at 6 months	16 (3%)	10 (3%)	6 (3%)	5 (12%)^***^	4 (20%)^***^
Complicated course^d^	205 (40%)	101 (32%)	104 (52%)^***^	35 (81%)^***^	18 (90%)^***^
False-positive LF-LAM test^e^	97 (19%)	–	97 (48%)	8 (19%)	2 (10%)

All values represent absolute number (%), or median (interquartile range)

* *P* < 0.05, ** *P* <0.01, *** *P* ≤ 0.001 (comparisons using Kruskal–Wallis and χ^2^ tests at the different cut-offs)

^a^Cough, fever, weight loss or night sweats in last month

^b^One patient did not have a CD4 count recorded at enrolment; 103 (20%) patients did not have an available haemoglobin; 107 (21%) did not have an available white blood cell count; 110 (21%) did not have an available lymphocyte count; 111 (21%) did not have an available creatinine; 143 (28%) did not have an available platelet count; 33 (6%) patients did not have an available chest-X-ray report

^c^Does not include the patients prescribed anti-TB therapy for a microbiologically confirmed diagnosis

^d^Complicated course: death, hospitalisation, confirmed TB diagnosis or initiation of empirical anti-TB therapy in the 6 months of follow-up

^e^Positive LF-LAM test on enrolment, but no death, hospitalisation, confirmed TB diagnosis or initiation of empirical anti-TB therapy in the 6 months of follow-up

*LF-LAM* lateral flow lipoarabinomannan, *TB* tuberculosis

### Clinical course

No patient was lost to follow-up (Fig. 1). After 6 months, 54/517 (10%) had a confirmed TB diagnosis including eight (15%) with rifampicin resistance on Xpert MTB/RIF testing. All 54 patients with a confirmed TB diagnosis received anti-TB therapy; 123 (27%) of the remaining 463 patients received empirical anti-TB therapy during the study in the absence of a confirmed TB diagnosis. Sixteen (3%) of the 517 patients died during the study — 6/54 (11%) enrolled as inpatients and 10/463 (2%) enrolled as outpatients. Only 4/54 (7%) inpatients and 2/463 (0.4%) outpatients died within 8 weeks of enrolment. Of the 463 enrolled as outpatients, 23 (5%) were hospitalised during follow-up.

### LF-LAM test results and inter-rater reliability

All 517 patients had a LF-LAM test performed on enrolment — 201 (39%) had a positive test (≥ grade 1), 43 (8.3%) had ≥ grade 2 positive test and 20 (3.9%) had a ≥ grade 3 positive test. The photograph of the LF-LAM test could be read with confidence in 503/517 (97%). The qualitative result was the same as the initial assessment in 459/503 (91%, kappa coefficient 0.82) using a grade 1 cut-off to define a positive test.

### Characteristics of patients with a positive LF-LAM test

The patients with a positive LF-LAM test were more likely to be inpatients at enrolment (*P* < 0.001) or have TB symptoms (*P* = 0.01). They were more likely to have advanced immunodeficiency (CD4 T-cell count < 100 cells/mm^3^) (*P* = 0.003), a history of hazardous alcohol use (*P* < 0.001), a body mass index < 18 kg/m^2^ (*P* = 0.03) or a haemoglobin level < 10 g/dL (*P* = 0.02) (Table 1).

### Correlation between a positive LF-LAM test result and the subsequent clinical course

Patients with a positive LF-LAM test were more likely to have a confirmed TB diagnosis during follow-up than patients with a negative test (36/201 (18%) vs. 18/316 (6%), *P* < 0.001) and were more likely to have a complicated course (104/201 (52%) vs. 101/316 (32%), *P* < 0.001) (Table 1, Additional file 1: Table S1 and Additional file 2: Table S2). The 43/517 (8.3%) patients with a more strongly positive test (≥ grade 2) were more likely than patients with a negative or grade 1 result to have a confirmed TB diagnosis during follow-up (17/43 (40%) vs. 37/474 (8%), *P* < 0.001) and to have a complicated course (35/43 (81%) vs. 170/474 (36%), *P* < 0.001). There were 97/517 (19%) patients in the entire cohort with a false-positive LF-LAM test. Of these 97 patients, 89 (92%) had a grade 1 positive test (Table 1).

### Correlation between a negative LF-LAM test result and the subsequent clinical course

Patients with a negative LF-LAM test were less likely to have a confirmed TB diagnosis (NPV (95% CI), 94% (91–97)) or complicated course in the ensuing 6 months (NPV (95% CI), 68% (63–73)). However, the absence of TB symptoms on clinical history had a similar ability to exclude both endpoints (NPV (95% CI), 94% (91–96) for a confirmed TB diagnosis and 71% (66–76) for a complicated course, respectively).

### Utility of the LF-LAM test in symptomatic inpatients

**Fig. 4:**
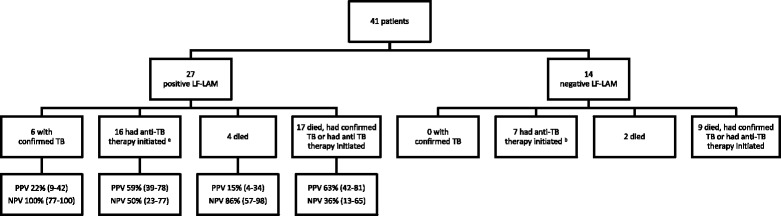
Ability of the LF-LAM test to predict important clinical endpoints in inpatients with TB symptoms. ^a^Includes the 6 patients with confirmed TB and 10 patients who had empirical anti-TB therapy started. ^b^All 7 patients had empirical anti-TB therapy started. *PPV* positive predictive value, *NPV* negative predictive value, both presented as percentage (95% confidence interval)

There were 41 (7.9%) inpatients in the cohort with symptoms of TB on enrolment (median CD4 T-cell count (IQR), 96 (37–277) cells/mm^3^) (Fig. 4). These patients were less likely to be receiving ART than the rest of the cohort (13/41 (32%) vs. 347/476 (73%), *P* < 0.001). Six (15%) of these 41 patients died; four had a positive LF-LAM test, three of whom received anti-TB therapy prior to death. Two of these three had TB diagnosed with Xpert on sputum, while the third had empirical anti-TB therapy for presumed TB meningitis. The one patient with a positive LF-LAM test who received no anti-TB therapy prior to death, died from laboratory-confirmed (India Ink positive) cryptococcal meningitis (Table 2). The LF-LAM test’s PPV in these 41 patients for either death, confirmed TB or empirical anti-TB therapy in the subsequent 6 months was 63% (95% CI, 42–81).

**Table 2:** Characteristics of the patients who died during follow-up

Age, Gender	CD4 T-cell count^a^	On ART^a^	On IPT^a^	Initial enrolment	LF-LAM result^a^	TB symptoms^b^	Anti-TB therapy before death^c^	Time of death^d^	Cause of death^e^
23, Female	56	Yes	No	Inpatient	Positive grade 3	Yes	Empirical	3 days	Suspected TB meningitis
32, Female	59	No	No	Inpatient	Positive grade 2	Yes	Directed	29 days	Confirmed TB meningitis
28, Female	14	No	No	Inpatient	Positive grade 3	Yes	Directed	34 days	Confirmed miliary TB
28, Female	144	Yes	No	Inpatient	Positive grade 4	Yes	No	87 days	Confirmed cryptococcal meningitis
33, Female	61	No	No	Inpatient	Negative	Yes	No	131 days	Suspected *Pneumocystis jirovecii* pneumonia
26, Female	53	Yes	No	Inpatient	Negative	Yes	No	3 days	Suspected *Pneumocystis jirovecii* pneumonia
48, Male	114	Yes	No	Outpatient	Positive grade 1	No	Empirical	63 days	Suspected miliary TB
36, Male	54	Yes	No	Outpatient	Positive grade 3	Yes	Empirical	79 days	Suspected TB meningitis
38, Female	121	Yes	No	Outpatient	Negative	No	Empirical	72 days	Suspected miliary TB
60, Female	96	No	No	Outpatient	Negative	No	Empirical	169 days	HIV related cachexia (died at home)
39, Male	512	Yes	No	Outpatient	Negative	No	Directed	69 days	Confirmed TB meningitis
34, Male	495	Yes	No	Outpatient	Negative	No	No	159 days	Confirmed cryptococcal meningitis
37, Male	39	Yes	No	Outpatient	Negative	No	Empirical	83 days	Suspected cerebral toxoplasmosis
31, Male	46	Yes	No	Outpatient	Negative	Yes	Empirical	82 days	Suspected TB meningitis
35, Female	107	Yes	No	Outpatient	Negative	No	No	27 days	Suspected cerebral toxoplasmosis
36, Male	80	No	No	Outpatient	Negative	Yes	Empirical	26 days	Suspected TB meningitis

^a^On enrolment

^b^Cough, fever, weight loss or night sweats in last month

^c^Three patients had directed therapy based on positive Xpert MTB/RIF assay on sputum

^d^Days after study enrolment

^e^Likely cause of death determined by attending clinician at time of death, unaware of LF-LAM result. Insufficient laboratory support to confirm diagnosis in most cases. Confirmed TB based on positive Xpert MTB/RIF assay of sputum. Confirmed cryptococcal meningitis based on India ink of cerebrospinal fluid. Suspected cerebral toxoplasmosis based on CT imaging results and clinical features. Suspected *Pneumocystis jirovecii* pneumonia based on clinical and radiological features

*ART* antiretroviral therapy, *IPT* isoniazid prophylaxis therapy *LF-LAM* lateral flow lipoarabinomannan, *TB* tuberculosis

Only 21/517 (4.1%) were inpatients with TB symptoms and a CD4 T-cell count of < 100 cells/mm^3^. These patients were less likely to be receiving ART than the rest of the cohort (7/21 (33%) vs. 353/496 (71%), *P* < 0.001). Five (24%) of the 21 patients died; three of whom had a positive LF-LAM test, but all received anti-TB therapy prior to death. Two of the three had TB diagnosed with Xpert on sputum, while the third had empirical anti-TB treatment for presumed TB meningitis (Table 2). The LF-LAM test’s PPV in these 21 patients for either death, confirmed TB or empirical anti-TB therapy in the subsequent 6 months was 83% (95% CI, 52–98).

### Ability of the LF-LAM test to avert mortality

Of the 16 deaths in the study, six occurred in inpatients and are described above. The other ten occurred in outpatients, eight of whom had a negative LF-LAM test. The remaining two patients both received empirical anti-TB therapy prior to death (Table 2).

## Discussion

In this observational study of consecutively enrolled HIV-positive Southeast Asian patients at a tertiary referral hospital, the LF-LAM test had limited clinical utility. The test had poor specificity and would have been unlikely to avert any deaths in the 6 months of the study. The test is also unable to identify drug resistance, which was present in almost 15% of the confirmed infections. In this Southeast Asian referral hospital setting at least, the LF-LAM test appears to add little to existing management strategies.

Most of the studies assessing the LF-LAM test have focussed on its sensitivity and specificity in diagnosing TB in HIV-positive patients in sub-Saharan Africa [4, 10]. However, the test’s PPV and NPV, which take into account the local prevalence of the disease, have more relevance to clinical decision-making [19]. The burdens of HIV and TB in Myanmar are among the highest in Southeast Asia: approximately 0.8% of adults in the country aged 18–49 are HIV-positive and TB kills an estimated 4800 HIV-positive patients annually [2, 22]. However, the incidence of HIV-TB co-infection in Myanmar is approximately 15 times lower than in South Africa, where much of the LF-LAM test’s validation has occurred [2, 23]. This is important to remember when considering LF-LAM-based diagnostic and management algorithms developed in Africa for use in other regions, as the test’s PPV for TB infection will necessarily be lower in countries with a lower TB prevalence [19].

In this series, the PPV of the LF-LAM test was poor — if clinicians had used the test to guide treatment, many patients would have received anti-TB therapy they did not require. For individual patients, this unnecessary therapy would have increased the risk of adverse effects and drug-drug interactions, and may have influenced adherence to ART [24, 25]. At a hospital level, there would have been the logistic and financial challenges of delivering this additional therapy [25]. While the test’s NPV was better, it was not superior to a focussed history in excluding the diagnosis, replicating the findings in another Southeast Asian cohort where a simple history was very helpful in excluding active TB [15].

Meanwhile, few, if any, of the deaths in the study would have been averted if the patients’ clinicians had been aware of the LF-LAM result. Only six of the patients who died in the study had a positive LF-LAM test, five of whom received anti-TB therapy before their death. The one patient with a positive LF-LAM result, who did not receive anti-TB therapy before death, died of laboratory-confirmed cryptococcal meningitis almost 3 months after enrolment. Despite the cohort’s median CD4 T-cell count of 270 cells/mm^3^, there were only six deaths in the first 8 weeks of the study, two of which occurred in outpatients. The low mortality rates seen in this study are similar to those seen from other studies performed in HIV-positive patients in Myanmar [26–28] and much lower than those seen in locations where the LF-LAM-based management algorithms have a mortality benefit [14].

WHO recommendations suggest that the LF-LAM test has greatest utility in hospital inpatients with signs and symptoms of TB and advanced immunodeficiency (CD4 T-cell count < 100 cells/mm^3^) [4]. Certainly, the test’s PPV for both a confirmed TB diagnosis and a complicated clinical course was greater in this group in this study (Additional file 1: Table S1 and Additional file 2: Table S2). However, the pre-test probability of a TB diagnosis in the ensuing 6 months in this population is already so high that any diagnostic test must have an excellent performance to affect the post-test probability and influence clinical management; unfortunately, in this series, that was not the case.

The poor specificity of the LF-LAM test in this cohort is quite different to the excellent specificity reported in some South African series with older versions of the test [4, 29]. However, we used the test according to the manufacturer’s instructions and we used the manufacturer’s reference card to define all positive tests. Independent review of photographs of the test strips excluded the possibility of significant test misinterpretation. The fact that over 90% of the false-positive tests were grade 1 positive results suggests a problem with using this cut-off to define a positive test. However, if the cut-off were raised to grade 2, the test’s sensitivity would fall and the false-positive rate would still be a concerning 19%.

It is unclear whether the poor specificity seen in this series is an issue with the test itself, the batch of tests that were used in this study, or the study doctors’ ability to read the tests. Even with the reference card, the fact that a consensus was sometimes required to interpret the test highlights some of the issues with its use in guiding clinical management and is reminiscent of the poor interobserver agreement when the faintest band was used to define a positive result in the older version of the test [21]. Cross-reactivity with non-tuberculous mycobacteria, including *M. kansasii*, *M. avium* and *M. fortuitum*, has also been proposed as an explanation for reduced specificity in different locations, and the prevalence of these organisms has substantial geographic variation [20, 30, 31].

Whatever the explanation for the disappointing results, they suggest that this novel diagnostic test is unlikely to have a major impact on TB-related mortality in HIV-positive patients in Myanmar. Meanwhile, established interventions that unequivocally reduce TB-related mortality remain poorly implemented in the country [28, 32]. There has been a significant recent increase in ART prescription in Myanmar [33]; however, even at this teaching hospital in the country’s largest city, almost one third of patients were not receiving ART at enrolment. Additionally, less than 3% of the patients in this cohort were receiving IPT despite its proven efficacy and recommendation in WHO guidelines [34–36].

Our results require validation. However, even if other studies in Asia showed the LF-LAM test to be more reliable, it is likely to have very limited everyday applicability in Myanmar [37]. The study took place at a high caseload referral hospital, but only 4% of the consecutively enrolled cohort were inpatients with symptoms of TB and advanced immunodeficiency, the population in whom the test is most helpful. With improving ART coverage in the country, the number of patients with advanced immunodeficiency is expected to fall, further reducing the test’s utility. Another under-reported limitation of the LF-LAM test is its inability to assist in the diagnosis of drug-resistant TB, present in 15% of this series and a significant and growing issue in Myanmar [2]. In any patient with a positive LF-LAM test, exclusion of drug-resistant TB would require supplementary testing.

The study’s interpretation is complicated by the frequent prescription of empirical anti-TB therapy [38]. However, while empirical anti-TB therapy has many limitations, in the absence of reliable diagnostic testing, its judicious administration in high-risk patients may sometimes be justified [25, 39]. Indeed, in a country where TB is the most common cause of death in HIV-positive patients, the mortality rate was low compared to other series from similar settings [40–43]. This does not necessarily suggest that empirical anti-TB therapy should be prescribed at the inflated rate seen here. However, the risk–benefit ratio of such a strategy can be optimised by targeting the patients at highest risk of disease and death, such as those with advanced immunodeficiency [44], while excluding patients at lower risk of disease such as those with recent treatment [45] or those with co-morbidities that might be exacerbated by drug toxicity [25]. Hopefully, future improvements in diagnostic testing for TB will eventually obviate the need for this relatively crude therapeutic approach.

The main limitation of the study is the fact that only sputum specimens were collected in the patients, a low-quality reference standard when extrapulmonary TB is common in HIV-positive patients [4]. Performing simultaneous mycobacterial culture of blood, urine and tissue specimens would have undoubtedly increased the number of confirmed TB diagnoses, but this requires substantial laboratory capacity that is not available in the public health systems of Myanmar and other Southeast Asian low- and middle-income countries. However, notwithstanding this fact, every patient was followed for 6 months after enrolment, permitting complete documentation of the important clinical endpoints of death, hospitalisation and the prescription of anti-TB therapy, and there were no patients lost to follow-up, a significant strength of the study. While the reference standard was imperfect, if a patient was alive without hospitalisation or receipt of anti-TB therapy in the 6 months of follow-up, it is reasonable to assume that the positive LF-LAM tests at enrolment in these patients were false positives. Indeed, our estimation of the false-positive rate is likely to be conservative as it is almost certainly the case that not all patients who were hospitalised or who received empirical anti-TB therapy had active TB.

## Conclusions

In this Southeast Asian tertiary referral hospital setting, the LF-LAM test generated a large number of false-positive tests, which, if used to guide therapy, would have resulted in many patients receiving unnecessary treatment. Although the test’s NPV was better, it was not superior to a simple, clinical history. Knowledge of the LF-LAM test result would have been unlikely to avert any of the deaths in the study and would not have recognised the significant number of drug-resistant cases. Even in the target population of inpatients with TB symptoms, a relatively small proportion of patients in even this high caseload hospital, the test had limited practical and clinical utility. Meanwhile, almost a third of patients at this tertiary referral hospital were not receiving ART at enrolment and 97% were not receiving IPT. While future studies may demonstrate that the LF-LAM test has utility in more nuanced, geographically specific, diagnostic algorithms, particularly in inpatients with advanced immunodeficiency, as a stand-alone test in this study, it added little to existing everyday diagnostic and management strategies. In the Southeast Asian setting, overcoming barriers to the rollout of ART and uptake of IPT are likely to have a far greater impact on reducing TB-related morbidity and mortality.

## Additional files

Additional file 1: Table S1. Performance of LF-LAM test in predicting a confirmed diagnosis of TB in sputum during 6 months of follow-up stratified by patient characteristics and the cut-off used to define a positive test. (DOCX 20 kb)

Additional file 2: Table S2. Performance of LF-LAM test in predicting a complicated course during 6 months of follow-up stratified by patient characteristics and the cut-off used to define a positive test. (DOCX 17 kb)

## Abbreviations

ART: antiretroviral therapy
IPT: isoniazid prophylaxis therapy
LF-LAM: lateral flow lipoarabinomannan
NPV: negative predictive value
PPV: positive predictive value
TB: tuberculosis
WHO: World Health Organization
