# Supplementary material for: The clinical utility of the urine-based lateral flow lipoarabinomannan assay in HIV-infected adults in Myanmar: an observational study
Source: BMC Med. 2017 Aug 4;15:145. doi: 10.1186/s12916-017-0888-3 (PMC5543584; doi:10.1186/s12916-017-0888-3)
Supplement: Supplementary file 1 — Performance of LF-LAM test in predicting a confirmed diagnosis of TB in sputum during 6 months of follow-up stratified by patient characteristics and the cut-off used to define a positive test. (DOCX 20 kb) [file 12916_2017_888_MOESM1_ESM.docx]

Table S1. Performance of LF-LAM test in predicting a confirmed diagnosis of TB in sputum ^a^ during six months of follow-up stratified by patient characteristics and cut-off used to define a positive test.

|  |  | Number | Sensitivity (95% CI) | Specificity (95% CI) | PPV  (95% CI) | NPV  (95% CI) |
| --- | --- | --- | --- | --- | --- | --- |
| All patients | Grade ≥1 | 201/517 | 51% (44-58) | 69% (63-74) | 52% (45-59) | 68% (63-73) |
|  | Grade ≥2 | 43/517 | 17% (12-23) | 97% (95-99) | 81% (67-92) | 64% (60-68) |
|  | Grade ≥3 | 20/517 | 9% (5-14) | 99% (98-100) | 90% (68-99) | 62% (58-67) |
| Inpatients | Grade ≥1 | 35/54 | 88% (47-100) | 39% (25-55) | 20% (8-37) | 95% (74-100) |
|  | Grade ≥2 | 15/54 | 75% (35-97) | 80% (66-91) | 40% (16-68) | 95% (83-99) |
|  | Grade ≥3 | 10/54 | 50% (16-84) | 87% (74-95) | 40% (12-74) | 91% (78-97) |
| Outpatients | Grade ≥1 | 166/463 | 63% (48-77) | 67% (62-72) | 17% (12-24) | 94% (91-97) |
|  | Grade ≥2 | 28/463 | 24% (13-39) | 96% (94-98) | 39% (22-59) | 92% (89-94) |
|  | Grade ≥3 | 10/463 | 11% (4-24) | 99% (97-100) | 50% (19-81) | 91% (88-93) |
| Symptomatic ^b^ | Grade ≥1 | 79/169 | 61% (51-70) | 75% (63-85) | 80% (69-88) | 54% (44-65) |
|  | Grade ≥2 | 32/169 | 27% (19-37) | 94% (85-98) | 88% (71-96) | 45% (36-53) |
|  | Grade ≥3 | 16/169 | 14% (8-23) | 98% (92-100) | 94% (70-100) | 42% (34-50) |
| CD4 ≥ 200 | Grade ≥1 | 110/324 | 42% (32-52) | 69% (63-75) | 37% (28-47) | 73% (67-79) |
|  | Grade ≥2 | 15/324 | 11% (6-19) | 98% (96-100) | 73% (45-92) | 72% (66-78) |
|  | Grade ≥3 | 4/324 | 3% (1-9) | 100% (98-100) | 75% (19-99) | 70% (65-75) |
| CD4 < 200 | Grade ≥1 | 90/192 | 58% (49-68) | 67% (56-77) | 69% (58-78) | 57% (47-67) |
|  | Grade ≥2 | 27/192 | 22% (14-31) | 95% (89-98) | 85% (66-96) | 50% (42-58) |
|  | Grade ≥3 | 15/192 | 13% (7-21) | 99% (94-100) | 93% (68-100) | 48% (40-56) |
| CD4 < 100 | Grade ≥1 | 55/102 | 64% (51-75) | 67% (48-82) | 80% (67-90) | 47% (32-62) |
|  | Grade ≥2 | 20/102 | 28% (17-40) | 97% (84-100) | 95% (75-100) | 39% (28-50) |
|  | Grade ≥3 | 11/102 | 16% (8-27) | 100% (89-100) | 100% (71-100) | 36% (26-47) |
| CD4 < 50 | Grade ≥1 | 28/50 | 70% (51-85) | 65% (41-85) | 75% (55-89) | 59% (36-79) |
|  | Grade ≥2 | 8/50 | 27% (12-46) | 100% (83-100) | 100% (63-100) | 48% (32-64) |
|  | Grade ≥3 | 6/50 | 20% (8-39) | 100% (83-100) | 100% (54-100) | 45% (30-61) |
| Symptomatic, ^b^ CD4 ≥200 | Grade ≥1 | 30/77 | 54% (37-69) | 78% (61-90) | 73% (54-88) | 60% (44-74) |
|  | Grade ≥2 | 8/77 | 15% (6-29) | 94% (81-99) | 75% (35-97) | 49% (37-62) |
|  | Grade ≥3 | 2/77 | 5% (1-17) | 100% (90-100) | 100% (16-100) | 48% (36-60) |
| Symptomatic, ^b^ CD4 <200 | Grade ≥1 | 48/91 | 65% (51-76) | 72% (53-87) | 83% (70-93) | 49% (33-65) |
|  | Grade ≥2 | 23/91 | 34% (22-47) | 93% (77-99) | 91% (72-99) | 40% (28-52) |
|  | Grade ≥3 | 13/91 | 19% (10-31) | 97% (82-100) | 92% (64-100) | 36% (25-48) |
| Symptomatic, ^b^ CD4 <100 | Grade ≥1 | 33/59 | 64% (49-78) | 71% (42-92) | 88% (72-97) | 38% (20-59) |
|  | Grade ≥2 | 17/59 | 38% (24-54) | 100% (77-100) | 100% (80-100) | 33% (20-50) |
|  | Grade ≥3 | 9/59 | 20% (10-35) | 100% (82-100) | 100% (66-100) | 28% (16-42) |
| Symptomatic, ^b^ CD4 <50 | Grade ≥1 | 19/32 | 68% (45-86) | 60% (26-88) | 79% (54-94) | 46% (19-75) |
|  | Grade ≥2 | 8/32 | 36% (17-59) | 100% (69-100) | 100% (63-100) | 41% (22-63) |
|  | Grade ≥3 | 6/32 | 27% (11-50) | 100% (69-100) | 100% (54-100) | 38% (20-59) |

^a^ Positive sputum culture or Xpert MTB/RIF assay (no extrapulmonary sampling was performed in the study).

^b^ Cough, fever, weight loss or night sweats in last month
