# Supplementary material for: The clinical utility of the urine-based lateral flow lipoarabinomannan assay in HIV-infected adults in Myanmar: an observational study
Source: BMC Med. 2017 Aug 4;15:145. doi: 10.1186/s12916-017-0888-3 (PMC5543584; doi:10.1186/s12916-017-0888-3)
Supplement: Supplementary file 2 — Performance of LF-LAM test in predicting a complicated course during 6 months of follow-up stratified by patient characteristics and the cut-off used to define a positive test. (DOCX 17 kb) [file 12916_2017_888_MOESM2_ESM.docx]

Table S2. Performance of LF-LAM test in predicting a complicated course ^a^ during six months of follow-up stratified by patient characteristics and cut-off used to define a positive test.

|  |  | Number | Sensitivity (95% CI) | Specificity (95% CI) | PPV  (95% CI) | NPV  (95% CI) |
| --- | --- | --- | --- | --- | --- | --- |
| All patients | Grade ≥1 | 201/517 | 67% (52-79) | 64% (60-69) | 18% (13-24) | 94% (91-97) |
|  | Grade ≥2 | 43/517 | 31% (20-46) | 94% (92-96) | 40% (25-56) | 92% (89-94) |
|  | Grade ≥3 | 20/517 | 17% (8-29) | 98% (96-99) | 45% (23-68) | 91% (88-93) |
| Inpatients | Grade ≥1 | 35/54 | 88% (47-100) | 39% (25-55) | 20% (8-37) | 95% (74-100) |
|  | Grade ≥2 | 15/54 | 75% (35-97) | 80% (66-91) | 40% (16-68) | 95% (83-99) |
|  | Grade ≥3 | 10/54 | 50% (16-84) | 87% (74-95) | 40% (12-74) | 91% (78-97) |
| Outpatients | Grade ≥1 | 166/463 | 63% (48-77) | 67% (62-72) | 17% (12-24) | 94% (91-97) |
|  | Grade ≥2 | 28/463 | 24% (13-39) | 96% (94-98) | 39% (22-59) | 92% (89-94) |
|  | Grade ≥3 | 10/463 | 11% (4-24) | 99% (97-100) | 50% (19-81) | 91% (88-93) |
| Symptomatic ^b^ | Grade ≥1 | 79/169 | 88% (71-96) | 63% (54-71) | 35% (25-47) | 96% (89-99) |
|  | Grade ≥2 | 32/169 | 44% (26-62) | 87% (80-92) | 44% (26-62) | 87% (80-92) |
|  | Grade ≥3 | 16/169 | 22% (9-40) | 93% (88-97) | 44% (20-70) | 84% (77-89) |
| CD4 ≥ 200 | Grade ≥1 | 110/324 | 48% (26-69) | 67% (61-72) | 10% (5-17) | 94% (90-97) |
|  | Grade ≥2 | 15/324 | 22% (7-44) | 97% (94-98) | 33% (12-62) | 94% (91-97) |
|  | Grade ≥3 | 4/324 | 9% (1-28) | 99% (98-100) | 50% (7-93) | 93% (90-96) |
| CD4 < 200 | Grade ≥1 | 90/192 | 80% (61-92) | 59% (51-67) | 27% (18-37) | 94% (88-98) |
|  | Grade ≥2 | 27/192 | 37% (20-56) | 90% (84-94) | 41% (22-61) | 88% (83-93) |
|  | Grade ≥3 | 15/192 | 20% (8-39) | 94% (90-97) | 40% (16-68) | 86% (81-91) |
| CD4 < 100 | Grade ≥1 | 55/102 | 89% (67-99) | 54% (43-65) | 31% (19-45) | 96% (85-99) |
|  | Grade ≥2 | 20/102 | 53% (29-76) | 88% (79-94) | 50% (27-73) | 89% (80-95) |
|  | Grade ≥3 | 11/102 | 26% (9-51) | 93% (85-97) | 45% (17-77) | 85% (76-91) |
| CD4 < 50 | Grade ≥1 | 28/50 | 100% (54-100) | 50% (35-66) | 21% (8-41) | 100% (85-100) |
|  | Grade ≥2 | 8/50 | 67% (22-96) | 91% (78-97) | 50% (16-84) | 95% (84-99) |
|  | Grade ≥3 | 6/50 | 50% (12-88) | 93% (81-99) | 50% (12-88) | 93% (81-99) |
| Symptomatic, ^b^ CD4 ≥200 | Grade ≥1 | 30/77 | 70% (35-93) | 66% (53-77) | 23% (10-42) | 94% (82-99) |
|  | Grade ≥2 | 8/77 | 30% (7-65) | 93% (83-98) | 38% (9-76) | 90% (80-96) |
|  | Grade ≥3 | 2/77 | 10% (0-45) | 99% (92-100) | 50% (1-99) | 88% (78-94) |
| Symptomatic, ^b^ CD4 <200 | Grade ≥1 | 48/91 | 95% (76-100) | 60% (48-72) | 42% (28-57) | 98% (88-100) |
|  | Grade ≥2 | 23/91 | 48% (26-70) | 81% (70-90) | 43% (23-66) | 84% (73-92) |
|  | Grade ≥3 | 13/91 | 24% (8-47) | 89% (79-95) | 38% (14-68) | 79% (69-88) |
| Symptomatic, ^b^ CD4 <100 | Grade ≥1 | 33/59 | 93% (68-100) | 57% (41-72) | 42% (25-61) | 96% (80-100) |
|  | Grade ≥2 | 17/59 | 60% (32-840 | 82% (67-92) | 53% (28-77) | 86% (71-95) |
|  | Grade ≥3 | 9/59 | 27% (8-55) | 89% (75-96) | 44% (14-79) | 78% (64-84) |
| Symptomatic, ^b^ CD4 <50 | Grade ≥1 | 19/32 | 100% (48-100) | 48% (29-68) | 26% (9-51) | 100% (75-100) |
|  | Grade ≥2 | 8/32 | 80% (28-99) | 85% (66-96) | 50% (16-84) | 96% (79-100) |
|  | Grade ≥3 | 6/32 | 60% (15-95) | 89% (71-98) | 50% (12-88) | 92% (75-99) |

^a^ Complicated course: death, hospitalisation, confirmed TB diagnosis or initiation of empirical anti-TB therapy. Chosen as an endpoint to capture those patients who may have had extrapulmonary TB.

^b^ Cough, fever, weight loss or night sweats in last month
